# Pumping Venom: Valvilli Architecture in *Diacamma* (Hymenoptera: Formicidae) and Implications for Stinger Functionality

**DOI:** 10.1002/jmor.70174

**Published:** 2026-09-15

**Authors:** Alexandre Casadei‐Ferreira, Johan Billen, Sebastian Büsse, Julian Katzke, Riou Mizuno, Adrian Richter, Evan Economo

**Affiliations:** ^1^ Biodiversity and Biocomplexity Unit Okinawa Institute of Science and Technology Graduate University Onna Okinawa Japan; ^2^ Department of Cytology and Evolutionary Biology Zoological Institute and Museum University of Greifswald Greifswald Germany; ^3^ Department of Biology Bioscience Centre, KULeuven Heverlee Belgium; ^4^ Department of Entomology National Museum of Natural History, Smithsonian Institution Washington DC USA; ^5^ Faculty of Agriculture Kagawa University Miki Kagawa Japan; ^6^ Senckenberg Leibniz Institution for Biodiversity and Earth System Research Frankfurt Germany; ^7^ Department of Entomology University of Maryland College Park Maryland USA

**Keywords:** ants, cuticle biomechanics, material composition, sting actuation, venom pump

## Abstract

The insect stinger is one of the best‐known animal venom‐delivery structures. Although its internal structure is crucial for venom delivery, key components remain poorly understood. The valvilli are a pair of articulated structures within the valve chamber that act as ‘flaps’ and have often been overlooked or described only superficially. Here, using *Diacamma* nr. *indicum* as a model system, we combine high‐resolution micro‐computed tomography (micro‐CT), histological sectioning, serial block‐face scanning electron microscopy (SBF‐SEM) and confocal laser scanning microscopy (CLSM) to characterise the fine‐scale morphology and cuticular material composition of ant valvilli. We report structural differentiation within the valvilli, including distinct cuticular zones that likely correspond to different functions. CLSM imaging particularly highlights variation in sclerotisation, resilin distribution and ultrastructure, indicating that the valvilli are not uniformly flexible elements but rather complex, only partially deformable structures. SBF‐SEM of the distal lobe margin corroborates this material differentiation at the ultrastructural level, revealing a laterally continuous sublayer of regularly spaced, circular domains with reduced electron density, consistent with a periodically organised transition in cuticle composition or architecture at the procuticle level. Furthermore, comparative micro‐CT scans of stingers fixed in different actuation states indicate the involvement of a non‐antiphasic pattern of valvilli movement during stinger deployment, challenging the current understanding of valvilli‐assisted pumping based on rhythmic, alternating movements, as inferred from European honeybees. These findings offer new anatomical insight into the architecture of the ant stinger and provide a refined morphological basis for future studies of venom delivery in Hymenoptera. By clarifying how such delivery systems have been fine‐tuned over the evolution of a hyper‐diverse insect group, they may also inform the design of bio‐inspired micro‐scale injection and pumping systems.

## Graphical abstract

The insect stinger is among the best‐known venom‐delivery structures, yet its valvilli—paired flaps within the valve chamber—remain poorly understood. Using micro‐CT, histology, serial block‐face SEM and CLSM in the ant *Diacamma* nr. *indicum*, we show that the valvilli are not uniformly flexible flaps but are materially differentiated structures with distinct cuticular zones and graded sclerotisation and resilin. Micro‐CT of stingers across actuation states indicates a non‐antiphasic pattern of valvillus movement, challenging the rhythmic pumping model inferred from honeybees.

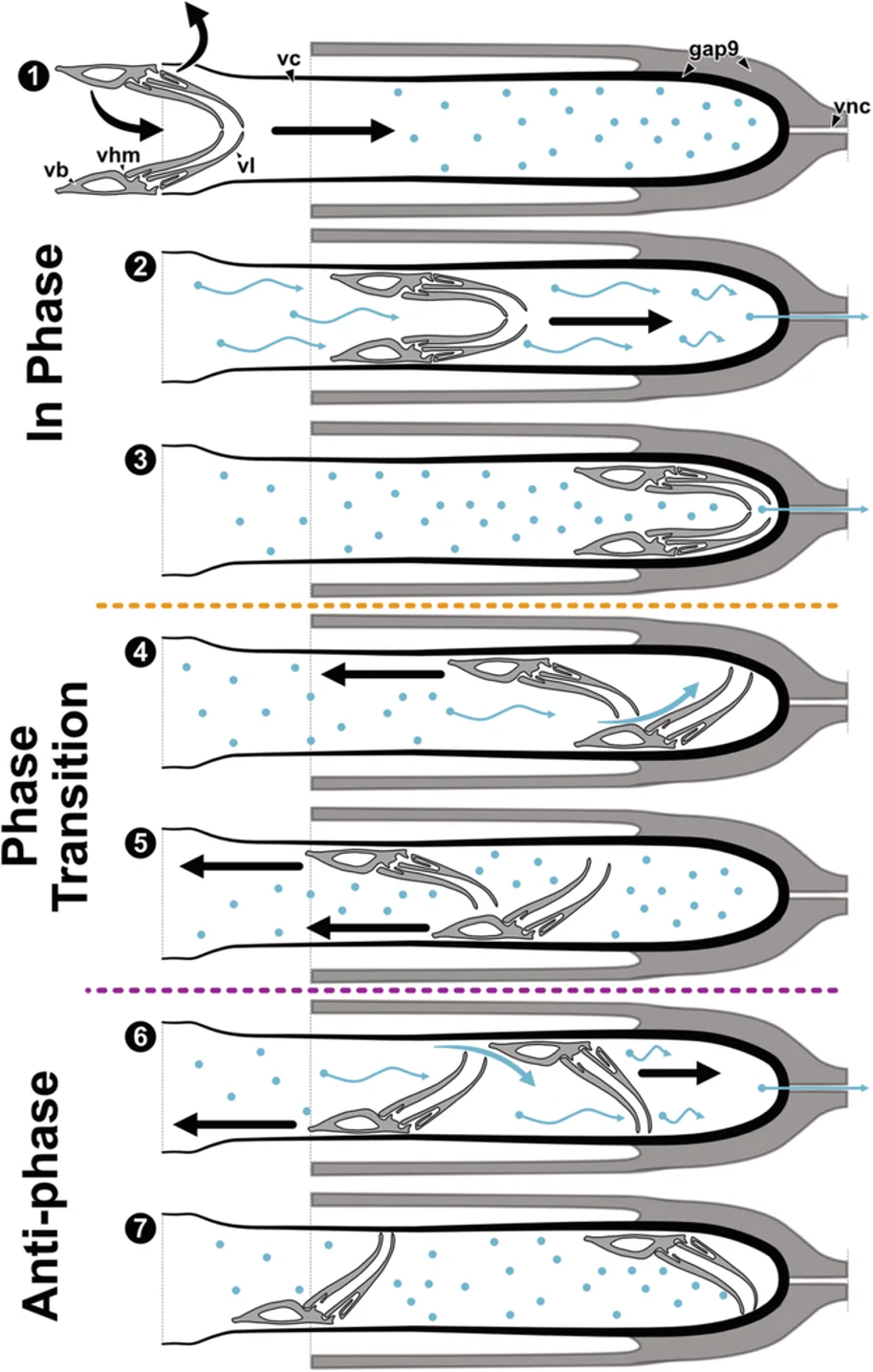

## 1 Introduction

Venom injection in Hymenoptera encompasses a diversity of morphological solutions, each evolved to achieve the critical biological goal of delivering venom effectively. Despite the apparent simplicity of venom injection through a sharp, pointed structure (Snodgrass 1933, 1956, 1983; Morse and Snodgrass 2018), close examination reveals substantial variation in the underlying biomechanical strategies among different hymenopteran lineages. While previous research has investigated various aspects of external stinger morphology (e.g., Hermann and Chao 1983; Kugler 1979; Kumpanenko et al. 2022; Kumpanenko and Gladun 2024, 2018; Lieberman et al. 2022; Ramirez‐Esquivel and Ravi 2023; Snodgrass 1933, 1956; Stetsun et al. 2019; Stetsun and Matushkina 2020) and the biochemical composition of venom (e.g., Arnaud Touchard et al. 2015; Axel Touchard et al. 2016), the mechanisms of venom delivery remain poorly understood. This is particularly true in taxa where differentiated internal structures modulate venom pumping. In these cases, structural and functional relationships are unclear.

Within Aculeata (ants, bees and wasps), two fundamental mechanisms for venom injection have been described: ‘pumping’ and ‘injection’ (Van Marle and Piek 1986; Stetsun and Matushkina 2020). The pumping mechanism, characteristic of bees (Apoidea) and ants (Formicidae), involves specialised internal structures known as valvilli (Lieberman et al. 2022; Snodgrass 1933). These flap‐like structures, positioned on the lancets (Lieberman et al. 2022, stinger parts that form the venom channel), move rhythmically during venom delivery, generating hydraulic pressure that drives venom posteriorly through the venom canal (Ramirez‐Esquivel and Ravi 2023; Snodgrass 1933). Although this pumping mechanism has been characterised only in bees and ants, the valvilli themselves are more widely distributed across Aculeata, occurring also in Crabronidae (Stetsun 2020), Mutillidae and Myrmosidae (Kumpanenko et al. 2022) and in Chrysidoidea, where their structure differs from that of other aculeates (Oeser 1962). Whether they serve the same pumping role in these groups remains unknown.

Notably, despite the widespread use of the terms ‘valves’ or ‘valvilli’ to describe these internal structures, a recent study (Ramirez‐Esquivel and Ravi 2023) argues that their functional role in venom injection is more similar to that of collapsible pistons than to true valves. Ramirez‐Esquivel and Ravi (2023) argue that the apparatus regulates directionality without generating force, whereas pistons actively create positive pressure to propel fluid. Consequently, the term ‘valve’ here arises from morphological homology rather than strict functional analogy, introducing potential confusion when discussing the biomechanics of venom injection. However, for consistency with existing literature, we continue to use the term ‘valvilli’ throughout this study (Snodgrass 1956; Lieberman et al. 2022).

In contrast to the pumping mechanism of ants and bees, most wasps (i.e., Vespidae and Pompilidae) employ an injection‐type mechanism that lacks valvilli (Van Marle and Piek 1986; Stetsun and Matushkina 2020; Kumpanenko and Gladun 2018, 2024). In these groups, the venom duct opens in the posterior region of the bulb, and venom is ‘squeezed’ entirely by musculature‐driven hydraulic pressure around the venom reservoir and duct (Van Marle and Piek 1986). Interestingly, several wasp families, such as Mutillidae (velvet ants) (Kumpanenko et al. 2022), possess valvilli‐like structures (Gadallah and Assery 2004; Kumpanenko et al. 2022) resembling those found in bees and ants, although the extent to which these contribute to venom pumping remains unclear. This variation suggests that the presence of valvilli and the use of pumping versus injection mechanisms do not follow a simple phylogenetic pattern and represent diverse evolutionary solutions to venom delivery.

While the ‘pumping’ and ‘injection’ mechanisms are often presented as distinct and mutually exclusive systems, Robertson (1968) noted that in some bees, scoliids and thynnids, a small but distinct muscle remains associated with the venom reservoir; a comparable muscle has since been reported across several ant lineages, including the ponerine *Brachyponera* (Billen and Al‐Khalifa 2018), multiple Leptanillinae (*Leptanilla*: Hölldobler et al. 1989; Billen et al. 2022); *Protanilla* (Billen et al. 2013); *Opamyrma*: (Yamada et al. 2020), *Myrmoteras* (Billen et al. 2015), *Camponotus* (Zhou et al. 2018) and several Dolichoderinae (Billen 1986a), indicating that the retention of this musculature is phylogenetically widespread rather than exceptional. Although this muscle is far less developed than the strong musculature that encases the reservoir sac found in injection‐type wasps, its presence suggests that venom ejection in these taxa may not rely solely on pressure generated by the valvilli. This indicates the possibility of intermediate or mixed systems within Aculeata, in which valvilli motion and residual glandular musculature contribute to venom injection. Such arrangements could represent retained ancestral traits or transitional stages in the evolutionary shift between injection‐type and pumping‐type stingers, potentially with individual adaptive benefits and trade‐offs.

In addition to the functional diversity of venom injection strategies, the morphological characteristics of valvilli themselves show considerable variation across Hymenoptera (Quicke et al. 1992). Although valvilli are generally articulated pairs of chitinous flaps on the lower ovipositor or stinger valves, their number, position and structural complexity vary widely (Quicke et al. 1992). Detailed surveys using light and electron microscopy have identified valvilli in most Ichneumonoidea and various aculeate lineages, whereas they are absent from other groups, including Symphyta, Evanioidea and Cynipoidea (Quicke et al. 1992). Among Aculeata, valvilli typically appear as complex, paired structures mounted on a distinct base, an arrangement structurally adapted to facilitate venom pumping (Snodgrass 1956; Ramirez‐Esquivel and Ravi 2023; Kumpanenko et al. 2022), though a simpler, single‐flapped valvillus occurs in several crabronid apoid wasps (*Oxybelus uniglumis*, *Lestica alata* and *Crabro scutellatus*; Stetsun 2020)—whether this reduction has any functional consequence for venom delivery remains unresolved. By contrast, in parasitoid Ichneumonoidea, valvilli are structurally simpler and lack a defined base, likely reflecting their primary function in egg laying rather than venom injection (Quicke et al. 1992). Phylogenetically, the distribution and structural variation of valvilli appear significant, especially within Ichneumonidae and Braconidae, where differences in valvillus morphology correlate closely with broader evolutionary relationships (Quicke et al. 1992). The morphological and functional diversity of valvilli raises intriguing questions about the evolutionary pressures that shaped these features, including their biomechanical roles in venom injection and oviposition. This emphasises the need for detailed anatomical and functional studies of valvilli across different hymenopteran groups to provide context for exploring their biomechanical roles in venom injection and oviposition systems.

Specifically, if muscle‐based venom ejection is energetically or mechanically efficient, what evolutionary pressures favoured the development of valvilli in ants, bees and some wasps? Conversely, if the valvilli‐driven mechanism optimises a parameter of venom injection (e.g., dosage control, resistance to backflow or sustained performance over repeated use), why have many wasp lineages developed a muscle‐driven system? One possibility is that muscle‐driven ejection enables faster, high‐volume discharge, well‐suited to rapid defence or prey subjugation, whereas valvilli pumping enables finer control, consistency or pressure regulation across multiple stings. Answering these questions will require detailed comparative analyses of internal stinger morphology, the material properties of the cuticle and an understanding of the underlying fluid‐dynamic principles operating at microscopic scales in the different systems. Our current contribution aims to lay the groundwork for further exploration of these questions.

To date, most functional inferences about valvilli have assumed a simple ‘flap‐like’ structure capable of producing alternating movement to generate pressure for venom injection. However, no study has yet examined these structures using a combination of modern imaging techniques that reveal fine‐scale morphology, cuticular material differentiation and the relative positioning of the valvilli captured at successive stages of the stinging cycle, from which their movement can be inferred. Here, based on the workers of *Diacamma* nr. *indicum*, we examine the internal stinger morphology of ants in detail, combining high‐resolution micro‐computed tomography (micro‐CT) scanning, histological sectioning, serial block‐face scanning electron microscopy (SBF‐SEM) and confocal laser scanning microscopy (CLSM). Our goal is to characterise the three‐dimensional architecture and material composition of the valvilli cuticle in unprecedented detail. Our analyses reveal that valvilli deviate substantially from the assumed ‘flap‐like’ paradigm, exhibiting differentiated cuticular material composition, apparent structural interdigitation and movement patterns that are not strictly antiphasic, as suggested by specimens fixed at alternating phases of the sting cycle. These findings suggest that valvilli mechanics are more complex than previously recognised, with implications for how venom injection is understood across Hymenoptera more generally.

## 2 Materials and Methods

### 2.1 Specimens Acquisition

**Figure 1:**
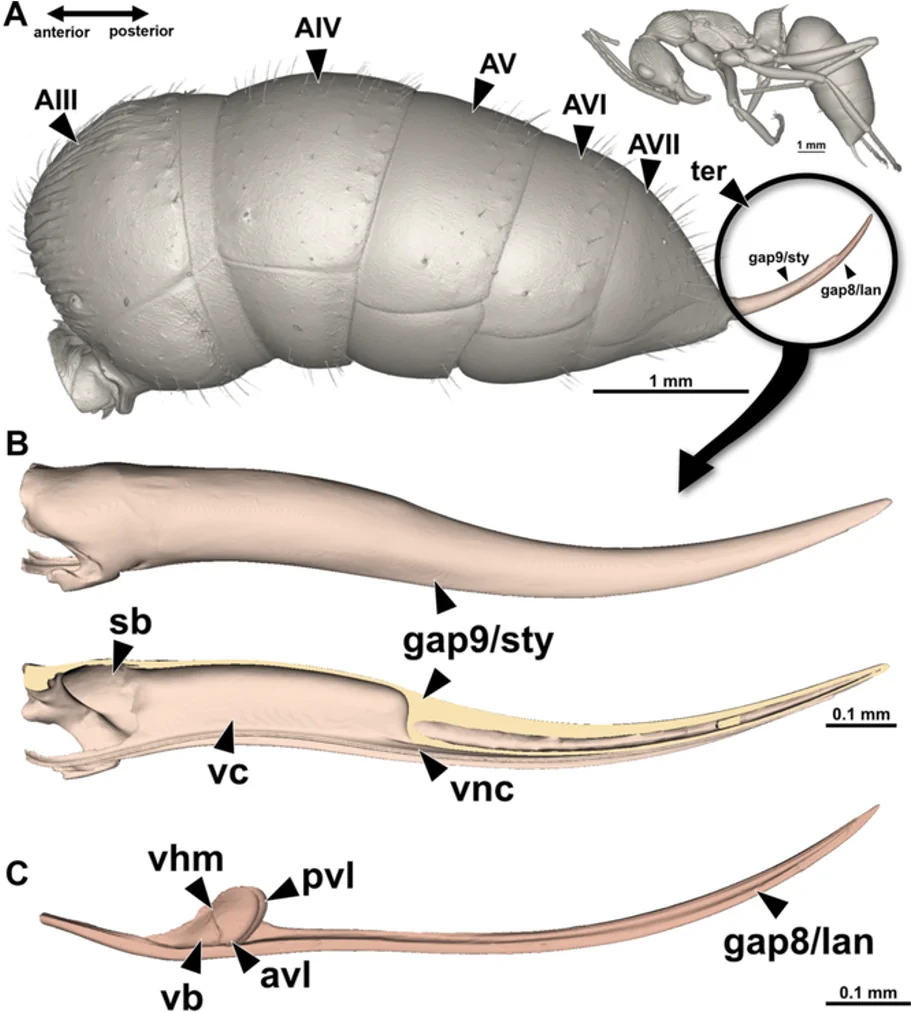
Three‐dimensional reconstruction of the stinger apparatus in *Diacamma* nr. *indicum*. (A) External habitus showing abdominal segments AIII–AVII and the terebra (*ter*). The full‐body 3D reconstruction was generated from micro‐CT data from the AntScan database (specimen ID: CASENT0745048; freely available at https://www.antscan.info; Katzke et al. 2026). (B) 3D reconstruction of the stinger, including the stinger base (*sb*), stylet (*gap9/sty*, gonapophyses IX) and valve chamber (*vc*). (C) Close‐up of a single lancet (*gap8/lan*, gonapophyses VIII) with associated internal structures: valvillus base (*vb*), hinge membrane (*vhm*), anterior (*avl*) and posterior (*pvl*) valvillar lobes and venom channel (*vnc*).

For comparative morphological analyses, we used freshly collected specimens of *D.* nr. *indicum* (Formicidae: Ponerinae) from a population in Okinawa, Japan. This approach ensured consistency in body size and morphology and was also motivated by the species’ status as a well‐established model for the study of ant behaviour and social biology (Tsuji 2021), as well as its robust stinger, capable of piercing relatively tough substrates, including human skin (R.M. pers. obs.). Workers (adults, body size 8–16 mm) were collected from Kenmin no Mori (Onna, Kunigami District, Okinawa; 26.5063°N, 127.9090°E) and maintained in laboratory nests to provide direct access to fresh material. To capture variation in stinger posture during venom injection, individuals were manually provoked to sting and then flash‐frozen with nitrogen spray. The resulting specimens had the stinger in either an extended (contracted/activated) or resting (relaxed) position (Figure 1A). Two individuals exhibiting clear, opposing stinger configurations were selected for high‐resolution micro‐CT. Additional individuals from the same colony were used for CLSM and SBF‐SEM. For both techniques, fresh specimens were dissected to isolate the stylet or lancet from the stinging apparatus and preserved in 99% ethanol until imaging. Specimens for histological sectioning were collected separately by J.B. in Okinawa and were not part of the main laboratory colony (see the Histology Section below for preparation details). Comparative analysis of internal stinger morphology among workers is based on micro‐CT data sets. Details of staining duration, scan settings and reconstruction parameters are provided in the Micro‐CT Scanning section.

### 2.2 Histological Sections

For histological examination of the stinger, the metasoma of three *D*. nr. *indicum* workers was carefully separated by transverse dissection between the 3rd (AIII) and 4th (AIV) abdominal segments. This approach facilitated effective penetration of fixatives and embedding media. Tissues were immediately fixed in cold 2% glutaraldehyde, buffered with 50 mM sodium cacodylate and supplemented with 150 mM saccharose to preserve structural integrity. Post‐fixation was performed with 2% osmium tetroxide (OsO_4_) prepared in the same buffer. Following fixation, specimens were dehydrated through a graded acetone series and embedded in Araldite epoxy resin. Serial semithin sections (1 μm thickness) were prepared on a Leica EM UC6 ultramicrotome (Leica Microsystems, Wetzlar, Germany) at the Zoological Institute, KU Leuven, Belgium. Sections were stained with a 0.1% methylene blue and thionin solution to enhance contrast for light microscopy and structural identification.

### 2.3 CLSM

To assess the material composition of the valvilli and adjacent regions of the stinger in *D*. nr. *indicum*, we employed CLSM on a Leica Stellaris 8 system (Leica Microsystems, Wetzlar, Germany) at the Zoological Collection of the University of Rostock, Germany. This technique leverages the autofluorescence of cuticular components to infer relative differences in mechanical and structural characteristics, such as sclerotisation and resilin content, based on cuticular material composition (after Michels and Gorb 2012). While this method allows qualitative mapping of relative material composition within the stinger, particularly the valvilli, it does not yield absolute quantitative values of the material properties of the respective cuticle. Interpretations of cuticle properties are therefore limited to relative fluorescence intensity and spatial distribution (e.g. Josten et al. 2022, for a critical discussion). Although CLSM has previously been used to image stinger structures (Stetsun et al. 2019; Stetsun and Matushkina 2020; Ma et al. 2023), this study represents, to our knowledge, the first application of the method targeting the valvilli.

Two dissected stingers were mounted using the ‘sandwich’ slide technique (Barbosa et al. 2014), in which the specimen is placed between two coverslips with play‐dough spacers to prevent deformation. A drop of pure glycerine was used as a mounting medium to preserve tissue flexibility during imaging. Autofluorescent signals were acquired using three laser excitation wavelengths selected to target different material classes within the cuticle. Sclerotised cuticle was excited at 633 nm, corresponding to rigid, highly cross‐linked chitin‐protein complexes. Flexible cuticle was excited at 488 nm, representing areas with intermediate mechanical properties. Resilin‐rich regions, associated with high elasticity, were excited at 405 nm (cf. Figure 1 in Büsse and Gorb 2018). Emitted light was detected in separate channels as grayscale images based on photon counts.

To maximise visual contrast and facilitate interpretation, we assigned each excitation channel a distinct false colour: cyan (405 nm, resilin‐dominated), yellow (488 nm, flexible cuticle) and magenta (633 nm, sclerotised cuticle). This CMY palette provides high visual discrimination without overlap or saturation, offering a more aesthetically coherent and colour‐blind‐friendly alternative to conventional RGB‐based schemes. Composite images were generated using ImageJ Fiji v1.54 (National Institutes of Health, USA).

### 2.4 Micro‐CT Scanning

High‐resolution X‐ray microtomography was performed on a Zeiss Xradia 510 Versa 3D X‐ray microscope, operated with the Zeiss Scout‐and‐Scan Control System, at the Imaging Section of the Okinawa Institute of Science and Technology Graduate University, Japan. Before scanning, seven specimens were stained in a 4% elemental iodine (I_2_) solution in 99% ethanol for 48 h to enhance soft‐tissue contrast. After staining, samples were rinsed in clean 99% ethanol for at least 12 h to remove excess iodine and standardise contrast conditions.

Scanning parameters were optimised for high absorption contrast and satisfactory resolution of internal structures. A low voltage of 40 kV was used to maximise contrast in iodine‐stained soft tissues. To improve edge definition and visualise subtle structural differences, the source–detector distance was slightly increased to enhance the system's inherent phase‐contrast capabilities. Each scan comprised 2001 X‐ray projections acquired over a full 360° rotation at ×4 and ×20 magnification. Exposure times were adjusted individually to produce approximately 4000 intensity counts in the most highly absorbing tissues, ensuring consistent brightness and signal across samples.

Scans were reconstructed using Zeiss software and exported for post‐processing. The reconstructed volumes were converted to NRRD format (‘*nearly raw raster data*’) and imported into 3D Slicer v5.8.1 (Brigham and Women's Hospital, USA) (Fedorov et al. 2012). 3D renders were generated using the *Colorize Volume* module of the 3D Slicer Sandbox extension for figure preparation.

### 2.5 Serial Block‐Face SEM

To characterise the fine‐scale cuticular architecture of the valvilli, the eighth gonapophyses (lancets) were processed for SBF‐SEM on a Teneo‐VS (Thermofisher Scientific, Waltham, MA, USA) at the Imaging Section, Okinawa Institute of Science and Technology Graduate University, Japan. A single specimen had been previously preserved in ethanol and was rehydrated in RO water before fixation in 2.5% glutaraldehyde buffered in PBS (pH 7.4). For contrast enhancement, samples were subjected to double osmium tetroxide staining, with the first incubation in a mixture of OsO_4_ and ferricyanide to enhance membrane contrast and improve conductivity, followed by a second OsO_4_ incubation. Sections were then stained with uranyl acetate. Specimens were dehydrated through a graded acetone series and embedded in Epon 812 resin (20 mL Epon 812, 10 mL DDSA, 10 mL MNA, 1 mL DMP‐30). Resin blocks were trimmed mechanically with progressively finer abrasives, with final trimming performed on an ultramicrotome equipped with a diamond knife. Prior to imaging, block faces were sputter‐coated with platinum/palladium using an MC1000 Ion Sputter Coater (Hitachi) to reduce charging artefacts. SBF‐SEM imaging was performed at an accelerating voltage of 2.8 kV, a beam current of 0.2 nA and a dwell time of 2 µs. Images were acquired at a pixel size of 4.76 nm (19.5 µm field of view across 4096 pixels). The microtome advanced 30 nm per cut, and images were collected every second section, yielding an effective Z‐voxel spacing of 60 nm. A total of approximately 2000 sections were acquired, corresponding to a cumulative imaging depth of ~50 µm through the valvillus region.

## 3 Results

**Figure 2:**
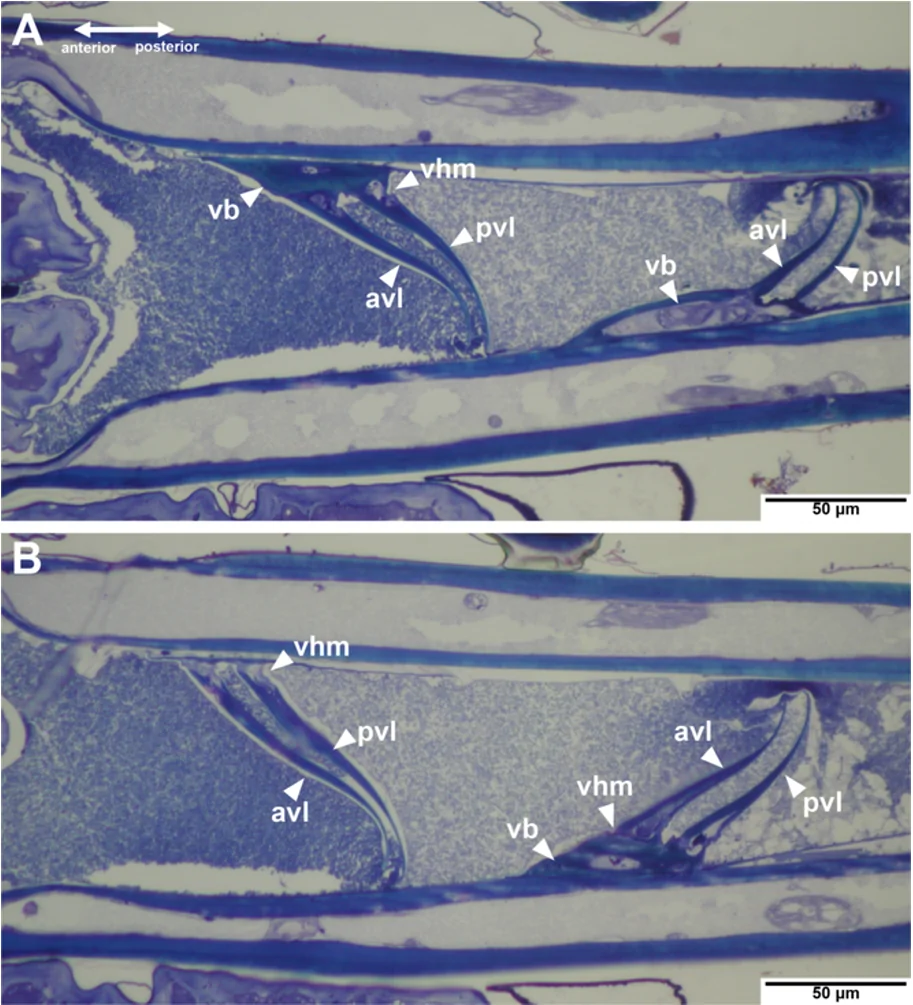
Coronal histological sections of the stinger showing valvillus organisation. (A, B) Histological sections through the stinger base of *Diacamma* nr. *indicum*, viewed in coronal (dorsoventral) orientation. Sections capture different dorsoventral levels: (A) more ventral; (B) more dorsal. Arrows indicate key components of the valvillus: valvillus base (*vb*), valvillus hinge membrane (*vhm*) and anterior (*avl*) and posterior (*pvl*) valvillar lobes.

In *D.* nr. *indicum*, the ninth gonapophyses are medially fused to form the distal part of the stinger base, comprising the bulb, the valve chamber (*vc*) and the stylet portion of the terebra (Figure 1B). The bulb and *vc* occupy the proximal region of the terebra, enclosing the space housing the venom‐pumping structures. The bulb forms the proximal chamber of this space, whereas the *vc* forms its distal continuation; the medially projecting sting bulb apophysis delimits the two chambers but does not fully septate their lumina, which therefore remain confluent (for detailed terminology and homology of the sting‐base chambers, see Lieberman et al. 2022). This chamber contains opposing valvilli that project medially from one of the paired lancets (eighth gonapophyses). In gross view, each valvillus can be divided into three parts: at its origin on the lancet is a stout basal section (here referred to as the valvillus base, *vb*; Figure 2), which is internally hollow and provides the main attachment to the cuticular wall of the lancet. Two elongated lobes extend distally from the base (anterior [*avl*] and posterior [*pvl*] valvillar lobes; Figure 2) and project into the *vc* towards the midline. The connection between the base and the lobes is formed by a flexible cuticular zone (valvillus hinge membrane, *vhm*; Figure 2) that allows the lobes to pivot or flex relative to the base. In situ, the paired valvilli face each other across the central lumen of the *vc*, occupying much of its dorsal space, with their lobes aligned so they can approach or slide past one another during stinger actuation. This arrangement positions the valvilli ideally for modulating the transfer of venom from the bulb into the venom canal and finally injecting it into the target.

**Figure 3:**
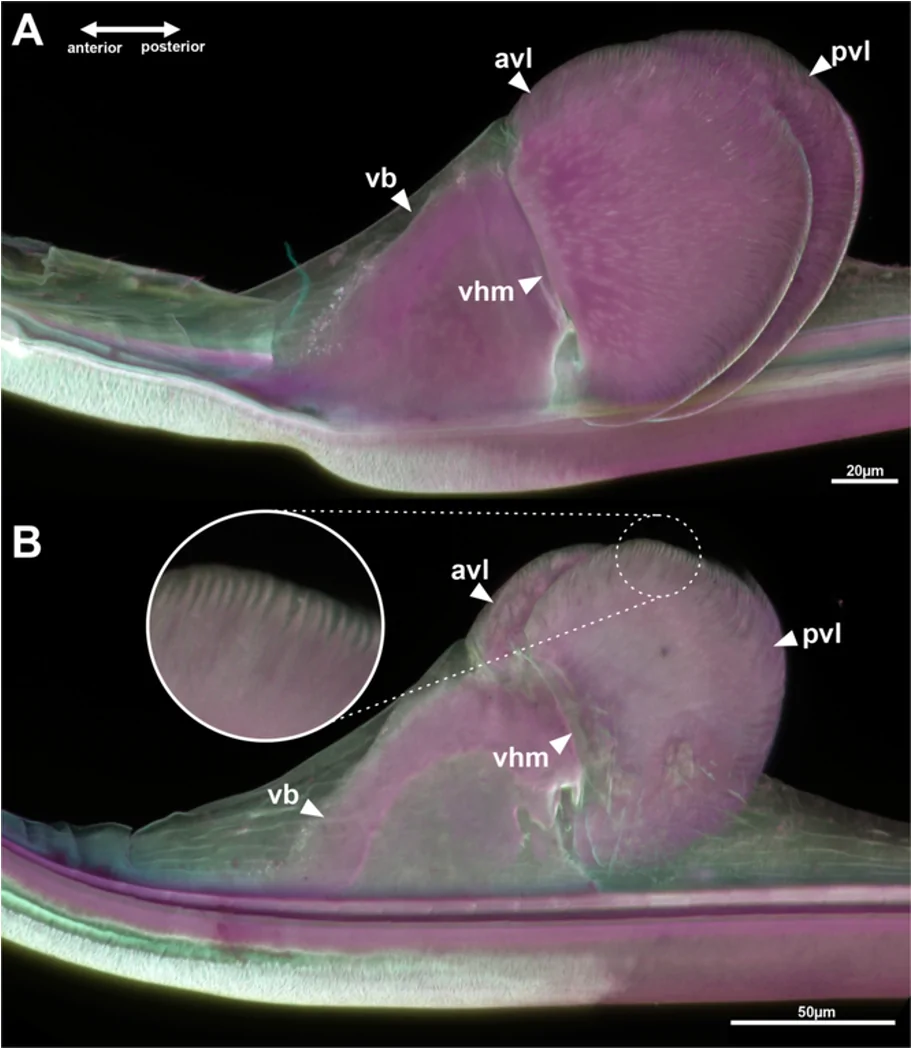
Material composition and internal structure of the valvilli, revealed by confocal laser scanning microscopy. (A, B) CLSM maximum‐intensity projections of the valvillus‐bearing region of the lancet in *Diacamma* nr. *indicum*, showing autofluorescence from sclerotised cuticle (magenta, 633 nm), flexible cuticle (yellow, 488 nm) and resilin‐dominated regions (cyan, 405 nm). (A) Median view showing the valvillus base (*vb*), hinge membrane (*vhm*) and anterior (*avl*) and posterior (*pvl*) valvillar lobes. The *vb* exhibits a strong magenta signal, indicating a rigid, sclerotised cuticle, while the *vhm* shows a mixed green signal (cyan + yellow), suggesting a flexible chitin–resilin composite. The lobes (anterior [*avl*] and posterior [*pvl*] valvillar lobes) are predominantly rigid but contain fine, longitudinally oriented internal bands of magenta signal extending distally from the hinge zone. (B) Lateral view at higher magnification of the distal lobe margin, revealing a segmented or ‘eyelash‐like’ edge where rigid elements (magenta) are embedded within a narrow band of flexible cuticle (green signal).

CLSM reveals distinct differences in the autofluorescence patterns of the stinger components, allowing qualitative assessment of the material composition of their cuticle (Figure 3 and Figure S1). The region of the lancet bearing the valvillus originates within a strongly sclerotised portion of the stylet wall (magenta signal; Figure 3), which extends into the basal section of the valvillus itself. The inferred stiffness of the *vb* is consistent with its structural role in anchoring the element to the lancet wall. In contrast, the *vhm* shows a mixed ‘green’ signal (Figure 3), interpreted as an overlap of cyan and yellow channels, indicating a flexible chitin–resilin composite that is likely more deformable than stiff sclerotised cuticle but less elastically responsive than resilin‐rich regions. The anterior (*avl*) and posterior (*pvl*) valvillar lobes are dominated by magenta (Figure 3), indicating a rigid composition. However, CLSM reveals a distinct internal organisation within the lobes, with fine, longitudinally oriented regions extending distally from the hinge membrane towards the apical margin (Figure 3). At higher magnification, the distal margin of the *avl* and *pvl* present a segmented or ‘eyelash‐like’ appearance, with rigid magenta elements embedded within a narrow band of mixed‐signal, more flexible cuticle (Figure 3B). The longitudinally oriented regions appear continuous with these marginal elements, producing a transition from the predominantly rigid central surface (core) of the lobe to a narrower flexible zone at its edge (Figure 3B). The two regions are differentiated by both colour signal and structural organisation.

**Figure 4:**
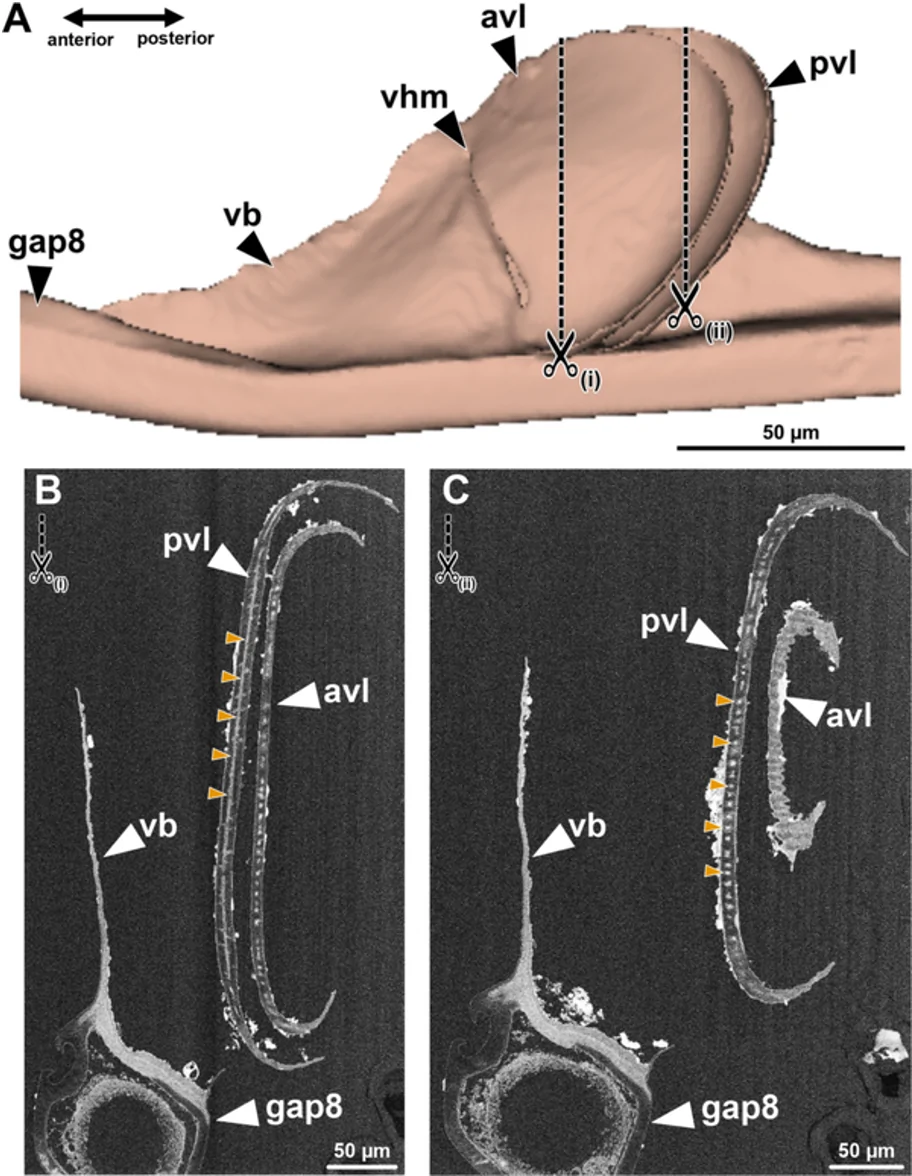
Ultrastructural organisation of the valvillar lobes revealed by serial block‐face scanning electron microscopy (SBF‐SEM). (A) Three‐dimensional reconstruction of the valvillus from micro‐CT data, showing the lancet (*gap8*), valvillus base (*vb*), valvillus hinge membrane (*vhm*) and anterior (*avl*) and posterior (*pvl*) valvillar lobes. Dashed lines with scissor symbols (i and ii) indicate the approximate cutting planes corresponding to the SBF‐SEM cross‐sections shown in (B) and (C). (B, C) Representative SBF‐SEM cross‐sections through the valvillus lobe at two cutting planes: (B) through the more proximal region of the lobe (plane i) and (C) through a more distal region (plane ii). In both sections, the outer cuticle layers display markedly higher electron density than the procuticle interior, consistent with the expected pattern of osmium staining. Small coloured (orange) arrowheads indicate a sublayer of reduced electron density within the valvillar lobes, proximally as a continuous sublayer corresponding to the rigid central surface (core) (Figure 3A,B), and distally as regularly spaced, circular domains that spatially correspond to the ‘eyelash‐like’ marginal zone identified by CLSM (Figure 3B). This periodically organised sublayer is present at both cutting planes, indicating its lateral continuity along the lobe margin.

SBF‐SEM of the valvilli revealed the expected pattern of heavy‐metal staining in cross‐section, with the outer cuticle layers showing markedly higher electron density than the procuticle interior (Figure 4B,C). Within the *avl* and *pvl*, however, a spatially discrete sublayer was apparent at the distal margin as a laterally continuous band containing regularly spaced, circular domains of reduced electron density embedded within an otherwise denser cuticular matrix (Figure 4B,C). These domains were uniform in apparent diameter and maintained consistent centre‐to‐centre spacing along the extent of the margin, giving the region a periodically structured appearance not seen in the bulk procuticle of the lobe or in the *vb*. This sublayer corresponds spatially to the zone identified by CLSM as the ‘eyelash‐like’ distal margin, where rigid elements are embedded within a narrow band of mixed‐signal flexible cuticle (Figures 3B and 4B,C). The coincidence of a compositionally distinct CLSM signal and a structurally distinct SBF‐SEM pattern at the same location suggests that this marginal zone differs from the surrounding cuticle in both material composition and ultrastructural organisation, though the precise nature of these domains, whether representing pore canals, compositional microdomains within the procuticle or another structural feature, cannot be resolved from the present data set alone.

**Figure 5:**
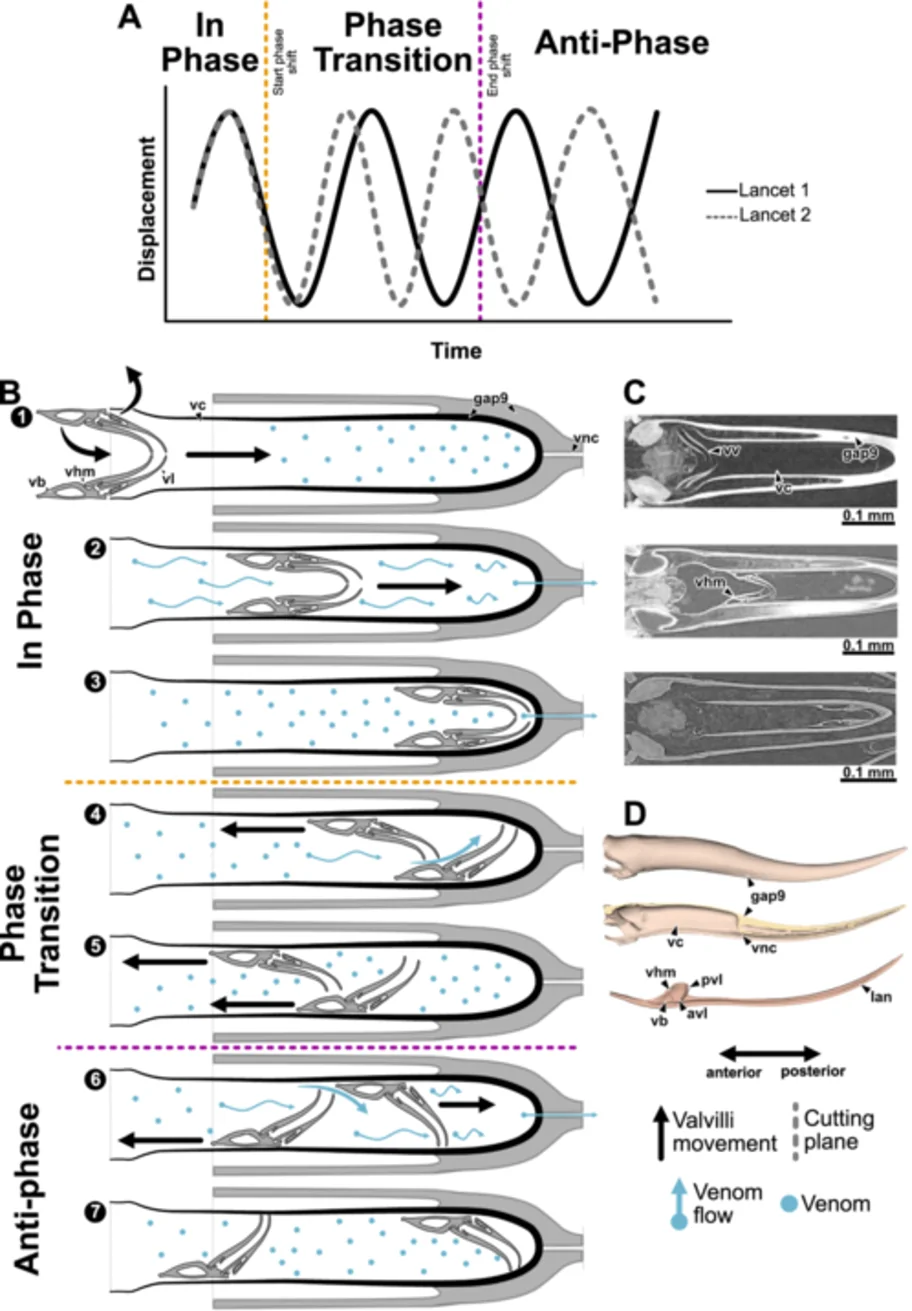
Proposed model of the stinging dual‐phase motion cycle in *Diacamma* nr. *indicum*. (A) Schematic representation of hypothesised phase transitions during a stinging cycle. The cycle begins with both lancets (and attached valvilli) moving distally in phase, compressing against the valve chamber. During the return stroke, the motion gradually shifts to an antiphasic pattern, enabling rhythmic valvillus‐based pumping of venom. (B) Illustration of valvilli mechanics in coronal section. When displaced in phase (1–3), the rigid lobes are deflected laterally and press against the chamber wall, forming a seal. In the transition stage (4–5), one valvillus retracts while the other remains advanced, allowing venom to refill the chamber and initiating the antiphasic stage (6–7). (C) Coronal micro‐CT slices of stingers fixed at different actuation states, all showing in‐phase configurations with paired valvilli (*vv*) displaced distally within the narrowed valve chamber. The hinge membrane (*vhm*) is not resolved but inferred from the gap between the valvillus base (*vb*) and anterior (*avl*) and posterior (*pvl*) valvillar lobes. Note variation in lobe orientation and chamber tapering along the distal‐proximal axis. (D) 3D reconstruction of the stinger, showing the stinger base (*sb*), stylet (*gap9*/*sty*, gonapophyses IX) and valve chamber (*vc*), and a detail view of a single lancet (*gap8*/*lan*, gonapophyses VIII) with associated valvillus structures, including base (*vb*), hinge membrane (*vhm*), anterior (*avl*) and posterior (*pvl*) valvillar lobes and venom channel (*vnc*). Collectively, the observed postures correspond to the two functional stages of the proposed dual‐phase pumping mechanism.

Micro‐CT scans of stingers fixed in different actuation states reveal substantial variation in the relative position and orientation of the paired valvilli within the *vc* (Figure 5C). In most reconstructions, the *vhm* was not clearly resolved and often appeared as a gap between the *vb*, *avl* and *pvl* (Figure 5C). The proximal bulb is consistently broader than the *vc*, which tapers distally towards the venom canal (Figure 5B,C). In three specimens, this narrowing coincides with a posture in which the valvilli are pressed closely together within the chamber. Their lobes deflect laterally towards the chamber wall due to the flexible *vhm*, which forms the articulation between the base and the lobes (Figure 5B,C). In this configuration, both valvilli might act together, as our micro‐CT images show them displaced distally (Figure 5C) and compressed against the distal wall of the chamber. The histological sections used in this study demonstrate a different posture (Figure 2), in which the valvilli are offset, with one displaced proximally while the other remains distal. This creates a visible gap between their medial margins. These two observed configurations (i.e., synchronous and asynchronous) correspond to the positional stages illustrated in the movement sequence diagram (Figure 5A,B; Movie S1).

## 4 Discussion

The internal pumping mechanism of the hymenopteran stinger has long been interpreted within a relatively narrow functional framework. Early foundational works (Snodgrass 1933; Van Marle and Piek 1986) and more recent analyses (Ramirez‐Esquivel and Ravi 2023) describe the valvilli as flap‐like cuticular projections that move in strict antiphasic motion to drive venom from the bulb into the venom canal. This view, largely based on observations of the European honeybee, has been widely applied to other Aculeata despite a lack of equivalent fine‐scale anatomical data. In particular, the structural complexity of the valvilli, especially the organisation of their lobes, remains undescribed, and no study has directly documented their configuration across different sting postures in a single species. Our combined micro‐CT, histological, SBF‐SEM and CLSM observations of *D.* nr. *indicum* reveal architectural and compositional variation not captured in previous models.

The cuticle of the *vb*, hinge membrane and lobes each show a distinct material composition—with gradients that lead to functionally distinct zones (Figures 2, 3, 4). The hinge area, which indicates a flexible, possibly resilin‐dominated zone, resembles a syndesis (i.e., a flexible, resilin‐rich connection between more rigid cuticular elements; see Josten et al. 2022) and may act as a pivot that permits repeated deflection of the rigid lobes on the *vb* during actuation. By contrast, the distal arrangement of the lobes terminates in a comb‐like edge, where rigid elements are embedded within a narrow, flexible band (Figure 3B). This arrangement and material transition suggest that the lobe margins may undergo localised deformation without a true joint, enabling them to seal against the chamber wall or slide past the opposing valvillus (Figure 5B,C). Additionally, SBF‐SEM of the distal lobe margin reveals a sublayer of regularly spaced, circular domains with reduced electron density, confined precisely to this zone (Figure 4B,C). This corroborates the CLSM‐derived evidence that the marginal band is compositionally and ultrastructurally distinct from the bulk lobe cuticle, whether through differential cross‐linking, heterogeneity in the protein–chitin matrix or the distribution of pore canals within this region.

In addition to these structural aspects, we discovered previously undocumented patterns in the relative positioning of the valvilli across stages of the stinging cycle. In contrast to previous studies on stinger movement, our observations show a synchronous configuration of the *Diacamma* stinging apparatus at the onset of the stinging process. Whereas a purely antiphasic movement pattern of the sting is assumed for bees and ants, our results indicate that *Diacamma*'s functional cycle may combine both synchronous and alternating phases. This potentially broader kinematic repertoire underscores the need to reassess pumping mechanisms across taxa and revisit long‐standing assumptions about stinger biomechanics in Hymenoptera. In more detail, histological sections (Figure 2) and micro‐CT imaging (Figure 5C) of stingers fixed in alternate postures suggest two discrete configurations, each consistent with a distinct phase of the pumping cycle: an offset arrangement consistent with antiphasic motion and an in‐phase stage. In the first, the valvilli adopt the expected offset arrangement (e.g., Ramirez‐Esquivel and Ravi 2023; Snodgrass 1933), in which their alternating displacement is thought to move venom distally through the canal (Figure 2). In the second, not previously reported in Aculeata with pumping mechanisms, both lancets are displaced distally, and their attached valvilli are compressed within the narrowed distal *vc* (Figure 5C), a configuration that would raise internal pressure and is therefore consistent with a venom‐expulsion stroke. This same arrangement of the valvilli—pressed together, with their lobes deflected laterally—was also observed in workers of *Acanthoponera*, *Pseudomyrmex* and *Stictoponera* (ACF, pers. obs.). Because those workers were fixed directly in 99% EtOH rather than frozen during stinger thrusting, we cannot determine whether the configuration represents a genuine actuation state or an artefact of fixation; only our *Diacamma* specimens, frozen at the onset of thrusting (three independently prepared specimens), document it securely as an early stage of the movement.

In this in‐phase posture of the lancets, the hinge membrane acts as a pivot, allowing the rigid lobes to deflect towards the chamber wall, bringing their medial margins into close contact and forming a seal (Figure 5C, Movie S1). The combination of a stiff core and a flexible margin likely enhances this seal by enabling precise conformity to the *vc*. Furthermore, the periodically organised sublayer identified in the SBF‐SEM signal (Figure 4B,C) suggests that this conformity is not solely a product of gross geometry but may reflect an architecturally patterned material transition at the procuticle level. This transition could optimise local deformability precisely where contact with the chamber wall or the opposing lobe occurs. Such a configuration supports two complementary mechanical actions. First, during the in‐phase stage (Figure 5A,C, Movie S1), the close apposition of the lobes within the narrowed distal *vc* may produce an effective seal, maximising pressure in the enclosed volume. This could drive the expulsion of a large bolus of venom in a single ‘push’ before the cycle transitions to an antiphasic phase (Figure 5A,C, Movie S1). Second, the flexible margins and embedded rigid elements (Figure 3B) may permit one lobe to deform and slide past the other during antiphasic motion without losing integrity, sustaining a continuous pumping cycle (functionality of phasic and antiphasic movement, see below). The structural discreteness of this marginal sublayer at the ultrastructural level (Figure 4B,C) further implies that this sliding capacity is enabled by localised cuticular material organisation rather than being a passive consequence of overall lobe geometry. Resolving the nature and mechanical role of these domains will require higher‐resolution imaging together with direct characterisation of the cuticle's material properties, both in its chemical composition (e.g., protein‐chitin content, degree of cross‐linking, metal deposit) and its local mechanical behaviour (e.g., stiffness measured by nanoindentation or comparable stress testing).

The presence of an in‐phase stage also has potential implications for the puncture mechanics of the stinger. As Anderson (2018) noted, puncturing ductile materials such as insect cuticle or vertebrate skin requires both fracture initiation and propagation. Delivering sufficient energy to initiate fracture is often the limiting factor, particularly for small‐mass organisms. In *Diacamma*, the in‐phase stage reflects synchronous movement of the paired lancets, so their sharpened tips advance together. This coordinated forward motion could transmit a concentrated mechanical load to the target, restricting deformation and facilitating fracture initiation (Anderson 2018, 2005; Atkins 2009; Blackman et al. 2013; Callister 2004). Once the fracture is formed, the system could transition to an antiphasic mode, in which alternating lancet movements propagate the puncture while sustaining venom delivery.

Alternating movements are well known in the ovipositors of parasitic wasps, which are homologous to the stingers of Aculeata (Cerkvenik et al. 2017; Eggs et al. 2018). In these taxa, push–pull mechanics between the individual valves enable insertion into resistant substrates while avoiding excessive axial loading that could cause buckling of the slender structure (Cerkvenik et al. 2017). Although the stylets of *Diacamma* are more robust than many ichneumonid ovipositors, an alternating anti‐phase stroke following in‐phase penetration could similarly reduce continuous axial force on the apparatus, lowering the risk of structural stress while maintaining forward progress. In ovipositors, differential protraction of the valves can also induce subtle steering of the tip by creating asymmetric reaction forces at the substrate interface (Cerkvenik et al. 2017). While such steering is unlikely to be a primary function in soft‐tissue stinging, alternating motion in *Diacamma* may nonetheless stabilise the puncture trajectory or compensate for minor lateral deflections during insertion. The underlying musculoskeletal arrangements that drive alternating motion in parasitoid wasps—antagonistic muscle pairs acting through lever‐like valvifers—are structurally conserved across Hymenoptera (Eggs et al. 2018) and likely underpin the rapid and coordinated transitions between synchronous and alternating phases (Figure 5A,C, Movie S1) that we hypothesised in *Diacamma*.

Within Aculeata, the sequence and coordination of stinger movements show notable variation, with both fully alternating and partly synchronous kinematics occurring. For the European honeybee, a strictly alternating sequence is described (Snodgrass 1933), in which the lancets advance and retract in turn, each anchoring via its barbs while the other moves forward (see Ramirez‐Esquivel and Ravi 2023 for further discussion). This ratcheting action progressively draws the stinger deeper with minimal resistance. Kumpanenko and Gladun (2018) documented a similar general pattern of alternating strokes in *Cryptocheilus versicolor* (Scopoli) (Pompilidae), but noted that specific structural characteristics, such as the wide anal arc of tergum IX and its articulation with the first valvulae, which creates a rigid connection between its two halves, can limit independent movement. This results in phases where both lancets advance or retract together. In other aculeate groups, including Vespidae, Formicidae and Apoidea, the anal arc is much narrower, which permits the halves of tergum IX to shift slightly against each other (Kumpanenko and Gladun 2018; Lieberman et al. 2022; Snodgrass 1956). This increased mobility enables the first valvulae to operate alternately in valve‑pump stingers. These morphological observations emphasise that stinger motion in Aculeata is not fixed to a single coordination pattern but instead reflects a continuum from fully alternating to partly synchronous sequences.

The dual‐phase coordination observed in *Diacamma*, combining distinct synchronous and alternating stages, adds another layer of complexity to this spectrum and highlights the adaptability of hymenopteran stinger kinematics. This mechanical flexibility also underpins how the in‐phase and anti‐phase stages will contribute differently to venom delivery. Movement patterns are likely to be directly related to the dynamics of venom pumping and flow. While the in‐phase stage plays a key role in fracture initiation and initial high‐pressure delivery, it also completely seals the *vc*, preventing refill. As we have not observed an opening between the valvilli in the in‐phase stage, an anti‐phase mode is essential to break the seal and consistently replenish the *vc* with venom. The rearward displacement of one lancet would create an opening between the lobes (Figure 5B, Movie S1), allowing venom to flow from the gland into the chamber. Thus, alternating back‐and‐forth movements might enable successive partial refills, maintaining a consistent supply to the venom canal.

The motoric complexity of the dual‐phase pumping cycle contrasts with the ‘injection’ mechanism found in many wasp lineages, where valvilli are absent and venom is discharged by direct muscular compression of the reservoir (Kumpanenko and Gladun 2024, 2018; Stetsun et al. 2019; Stetsun and Matushkina 2020; Van Marle and Piek 1986). Although this system is mechanically simpler and can deliver venom rapidly in large volumes, it sacrifices the fine metering and potential dual‐mode operation of a valve pump. The retention of valvilli in ants and bees may be favoured where controlled delivery is advantageous, for example, in delivering small amounts of venom to multiple targets, modulating venom output depending on context or maintaining pressure over extended injections (Haspel et al. 2003; Gnatzy et al. 2015; Haight and Tschinkel 2003). By contrast, the injection mechanism may be optimised in contexts where speed and volume outweigh precision, such as rapid defence or the subjugation of large prey (Haspel et al. 2003; Haight and Tschinkel 2003). Testing these ideas would require precise characterisation of venom delivery strategies in target species, as well as close behavioural observations of venom use, which are currently absent or limited for most relevant groups. However, the fact that some wasp families (e.g., Mutillidae; Kumpanenko et al. 2022) have retained valvilli suggests that selective pressures vary even within wasps, perhaps linked to prey handling, nesting biology or the balance between defensive and predatory functions of the stinger. Conversely, the loss of valvilli in other lineages may reflect a shift towards maximising delivery rate over control, reduced reliance on venom economy or morphological constraints that make the *vc* less effective.

The physical properties of the venom cocktail itself likely influence the efficiency of this refill‐and‐seal cycle. The physicochemical properties of ant venom and their implications for microscale flow are poorly characterised. Proteomic and peptidomic surveys (Touchard et al. 2016, 2015) have shown that ant venoms are complex cocktails comprising not only low‐molecular‐weight compounds and enzymes but also a broad range of peptides, from short linear cytolytic peptides to larger disulfide‐rich neurotoxins and dimeric structures. Many of these highly structured peptides may increase viscosity compared with simple aqueous solutions. Enzymatic components such as phospholipases, proteases and hyaluronidases, common in many ant venoms, can further alter local tissue environments, but their contribution to intra‐stinger fluid mechanics is unknown. No direct measurements of ant venom rheology exist, but studies on microneedle and microfluidic systems show that in channels narrower than ~100 µm, viscous drag dominates and surface tension can significantly resist liquid entry, especially for solutions containing larger biomolecules (Ita 2020; Martanto et al. 2005). Under such conditions, even moderate increases in viscosity can markedly slow passive flow, making active ‘opening’ phases crucial for rapid chamber refill. The venom apparatus further comprises a set of integrated glands whose morphology and function vary across Hymenoptera. The main venom gland produces the toxic secretion and delivers it via a venom duct. This process is often aided by a specialised filtering or narrowing structure that can regulate the flow of particles (Callahan et al. 1959). However, the closely associated Dufour's gland can function as a lubricant source and as a chemical adjunct to venom, in addition to producing pheromones involved in alarm signalling or trail marking (David Morgan 2009). In some taxa, notably certain species of ants, both the Dufour's and venom reservoir possess dedicated musculature capable of independently controlling secretion discharge, indicating that release is actively regulated rather than a passive by‐flow of stinging activity (Billen 1986b). In this context, it is important to view sting mechanics as part of an integrated system that couples glandular output, flow control and mechanical delivery.

While variation in stinger function is evident across aculeates, some of this inferred variation may stem from a limited understanding of the full systems in various species. European honeybee studies focus on the strict antiphasic cycles of piston‐like or collapsible valve‐like valvilli (Ramirez‐Esquivel and Ravi 2023; Snodgrass 1933; Van Marle and Piek 1986), yet this model may be incomplete. Our consideration of the in‐phase stage in *Diacamma* suggests it may confer advantages beyond the precision metering and continuous flow generated by the antiphasic stage. An in‐phase stage may occur in bees, but it could have been overlooked because previous studies either did not analyse stingers in multiple postures or used methodologies that precluded fine‐scale temporal resolution. Detecting such a phase would require targeted observation of stingers in different actuation states across a range of taxa. It remains unresolved whether the dual‐phase cycle in *Diacamma* reflects a lineage‐specific innovation or a shared capacity enabled by conserved morphological traits. To determine its distribution and evolutionary origin, comparative studies of valvilli architecture and sting kinematics are required across a wider phylogenetic range.

Although the *Diacamma* valvilli conform to the general aculeate body plan of a robust base and articulating lobes linked by a flexible hinge (Ramirez‐Esquivel and Ravi 2023; Snodgrass 1933; Van Marle and Piek 1986), the internal organisation of the lobes and the potential for dual‐phase venom delivery, as described here, have not been reported in any other hymenopteran. Quicke et al. (1992) treated the valvilli of Ichneumonoidea and Aculeata as homologous, yet the two differ markedly in position along the shaft (distal, near the ovipositor tip, in Ichneumonoidea vs. proximal, near the stinger base, in Aculeata), in structure (simple and confluent with the canal wall vs*.* paired and mounted on a distinct base), and in inferred function. Whereas the pumping role is well supported in Aculeata (Snodgrass 1956; Quicke et al. 1992; Ramirez‐Esquivel and Ravi 2023), the valvilli of parasitoid Ichneumonoidea have instead been hypothesised to hold the egg in position within the terebra, or to generate hydrostatic pressure that advances it towards the ovipositor tip (Rogers 1972; Quicke et al. 1992; Eggs et al. 2018), with the two roles not necessarily exclusive. Their equivalence, and with it the plesiomorphic mode of venom delivery in Apocrita, remains unresolved (Quicke et al. 1992). We therefore base our comparisons on functional criteria rather than strict homology. The single‐flapped valvilli of some crabronid apoid wasps (Stetsun 2020) represent an additional, untested axis of variation, and whether such reductions have mechanical consequences for venom pumping or instead reflect ecological or behavioural specialisation remains an open question worthy of further investigation.

Our findings also raise important questions about the evolutionary history of the pumping mechanism, for example: Did the in‐phase stage evolve within the Formicidae, or is it an ancestral condition of the pumping apparatus that has previously gone unnoticed? Is the fine‐scale compositional specialisation of the lobes a unique adaptation, or has it simply not been identified in other groups due to methodological limitations? By integrating structural, compositional and positional data, our study provides the most detailed morphological account of ant valvilli to date. It introduces a revised functional model that incorporates both in‐phase and anti‐phase dynamics (Figure 5 and Movie S1). The coupling of rigid and flexible elements within a single lobe, combined with dual‐mode motion, suggests that the ant valvillus apparatus is adapted to meet both the mechanical stresses during penetration and the physiological requirements of venom delivery. Directly resolving the kinematics of the pumping cycle would offer the most definitive test of these inferences, but it remains largely impractical in ants. The stinging apparatus in these animals is deeply internal, and although high‐speed X‐ray approaches such as synchrotron radiography or cineradiography (dos Santos Rolo et al. 2014; Betz et al. 2008; Schwyn et al. 2013) could, in principle, capture it in real time, doing so would require immobilising an individual within the beam's narrow field of view while simultaneously eliciting stinging behaviour, which, for now, is scarcely achievable. In the absence of such data, extending high‐resolution imaging and multi‐posture sampling to a broader range of taxa will be essential to determine whether this versatile pumping system is unique to ants, convergently evolved in other valvillus‐bearing lineages or ancestral to Aculeata and subsequently lost in some groups.

## 5 Conclusions

Our findings revise the functional paradigm of aculeate venom‑pumping mechanisms, departing from the canonical bee model, by showing that the *D.* nr. *indicum* stinger operates in a dual‑phase cycle, alternating between an in‑phase, high‑pressure stage and an anti‑phase, refill stage. High‑resolution micro‑CT imaging of stingers fixed in alternate postures provided direct morphological evidence of the in‑phase configuration, with both valvilli displaced distally and compressed together within the *vc*. This cycle is enabled by a previously undescribed internal organisation of the valvillar lobes, in which rigid structural support is coupled with locally deformable margins, balancing the mechanical demands of lancet‑driven penetration with the physiological requirements of sustained venom delivery. As the most detailed morphological account of ant valvilli to date, these results expand the known functional repertoire of the pumping mechanism and highlight the need for comparative, high‑resolution analyses across Hymenoptera to uncover the evolutionary origins and ecological drivers of this versatility. Targeted studies integrating in vivo kinematics with fine‑scale morphological and material analyses will be essential to determine whether this dual‑phase cycle is unique to ants or represents an overlooked ancestral condition within Aculeata with pumping mechanisms.

## Author Contributions

A.C‐F., J.K., R.M., A.R. and E.P.E. conceived the research. A.C‐F. processed the scan data, analysed the results and drafted the first manuscript version. J.B. processed the histological data. A.C‐F. and S.B. acquired CLSM images. J.K. acquired SBF‐SEM images. S.B., J.K. and A.R. supported data interpretation. R.M. supported fieldwork and maintained the laboratory colonies. A.C‐F., S.B., R.M., A.R. and E.P.E. procured funding. J.B., S.B., J.K., R.M., A.R. and E.P.E. contributed to the writing of the final manuscript and gave final approval before submission. E.P.E. supervised the research.

## Supporting information


Supporting File 1

Supporting File 2

## Data Availability

The data that support the findings of this study are available in the supporting material of this article.
