# Supplementary material for: Pumping Venom: Valvilli Architecture in Diacamma (Hymenoptera: Formicidae) and Implications for Stinger Functionality
Source: J Morphol. 2026 Sep 15;287(9):e70174. doi: 10.1002/jmor.70174 (PMC13576080; doi:10.1002/jmor.70174)
Supplement: Supplementary file 1 [file JMOR-287-e70174-s002.pdf]

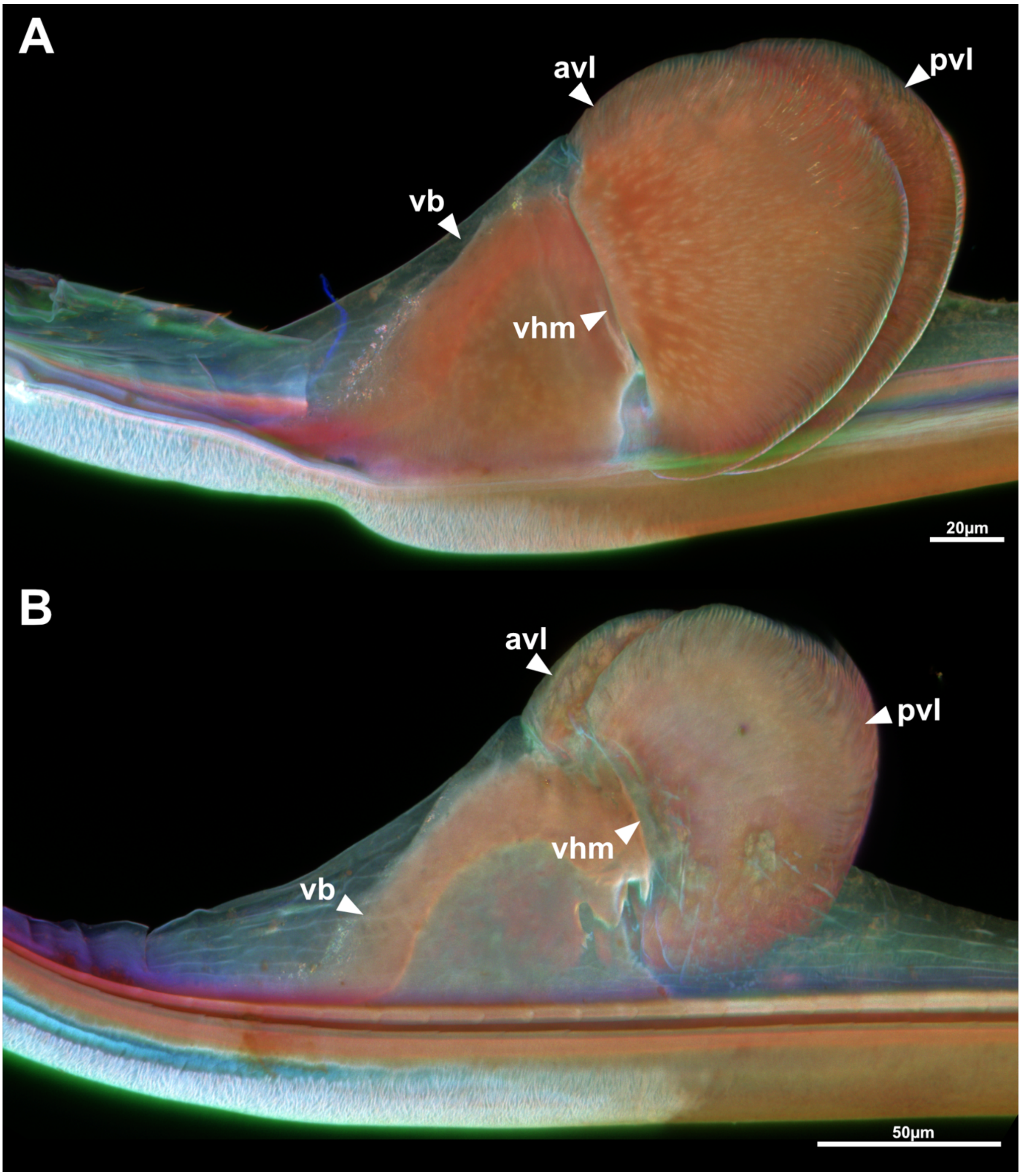

Supplementary Figure 1. (A, B) CLSM maximum-intensity projections of the valvillus-bearing region of the lancet in *Diacamma* nr. *indicum*, showing autofluorescence from sclerotised cuticle (red, 633 nm), flexible cuticle (green, 488 nm), and resilin-dominated regions (blue, 405 nm). (A) Median and (B) lateral view showing the valvillus base (*vb*), hinge membrane (*vhm*), and lobe (*vl*). The *vb* exhibits a strong red signal, indicating a rigid, sclerotised cuticle, while the *vhm* shows a mixed green signal (blue + yellow), suggesting a flexible chitin–resilin composite. The lobes (anterior (*avl*) and posterior (*pvl*) valvillar lobes) are predominantly rigid but contain fine, longitudinally oriented internal bands of red signal extending distally from the hinge zone.
